# Fatigue Reliability Assessment for Orthotropic Steel Decks Based on Long-Term Strain Monitoring

**DOI:** 10.3390/s18010181

**Published:** 2018-01-10

**Authors:** Yang Deng, Aiqun Li, Dongming Feng

**Affiliations:** 1 Beijing Advanced Innovation Center for Future Urban Design, Beijing University of Civil Engineering and Architecture, Beijing 100044, China; dengyang@bucea.edu.cn (Y.D.); liaiqun@bucea.edu.cn (A.L.); 2 Weidlinger Transportation Practice, Thornton Tomasetti, Inc., New York, NY 10005, USA

**Keywords:** orthotropic steel deck, structural health monitoring, fatigue reliability, gaussian mixture model, suspension bridge

## Abstract

A time-dependent fatigue reliability assessment approach is proposed for welded details of orthotropic steel decks (OSDs) using long-term strain monitoring data. The fatigue reliability limit function of the welded details is established based on the Eurocode specifications. Depending on the distribution characteristics of the measured daily equivalent stress range, either the lognormal distribution or Gaussian mixture model (GMM) is selected to quantify its uncertainty. Subsequently, the fatigue reliability can be calculated using either an explicit formula or the Monte Carlo method. This proposed approach is applied for the fatigue reliability evaluation of two rib-to-deck and two rib-to-rib welded fatigue details of an in-service suspension bridge. The results show that the reliability indices decrease significantly with bridge’s service life. Except for a rib-to-deck detail, all other three welded details cannot meet the target fatigue reliability during this bridge’s 100-year service life. The proposed approach can help bridge owners and operators make informed decisions regarding maintenance and repair of potential fatigue cracks.

## 1. Introduction

Orthotropic steel decks (OSDs) have been widely adopted for long-span bridges due to their notable advantages, such as light weight, high strength and durability, and rapid construction [1,2,3]. However, various types of cracking in the OSDs have been reported owing to lack of knowledge in its fatigue characteristics, design defects, and harsh loading conditions such as heavy-duty vehicles and high-density traffic volumes [1,4,5]. In the past few decades, significant efforts have been made on the design of S–N curves and development of fatigue life prediction approaches [6,7,8,9].

Fatigue reliability evaluation for the in-service OSD bridges requires accurate measurement of fatigue stress spectra, which can be obtained using the structural health monitoring system (SHMS) [2,10,11,12]. Existing SHMS-based fatigue evaluation approaches for the OSDs can be classified into two categories. The first type is to develop fatigue stress spectra using the finite element (FE) analysis based on measured operational vehicle flows by weigh-in-motion systems [12,13,14]. Because of complex structural behaviors and randomness in the actual traffic loadings, this type method relies on the accuracy of reconstructed vehicle loading models and developed FE models [12]. The second type is directly based on the strain measurements collected by strain sensors [10,11]. For example, Guo et al. [10] investigated effects of ambient temperature and traffic volume on fatigue damage of welded steel deck details using long-term strain monitoring data. Teixeira de Freitas et al. [11] applied strain measurements from controlled load tests and one-year of field monitoring to investigate the fatigue performance of an OSD strengthened by bonding a second steel plate to the existing deck. The above strain monitoring-based studies mainly focused on deterministic analyses.

In this study, to consider uncertainties in fatigue analysis, a reliability-based fatigue evaluation approach for welded details of orthotropic steel decks is proposed using both the fatigue strength curves from existing specification and the long-term monitoring strain data.

## 2. Formulation of the Proposed Fatigue Reliability Analysis

### 2.1. Fatigue Limit State Function

**Figure 1:**
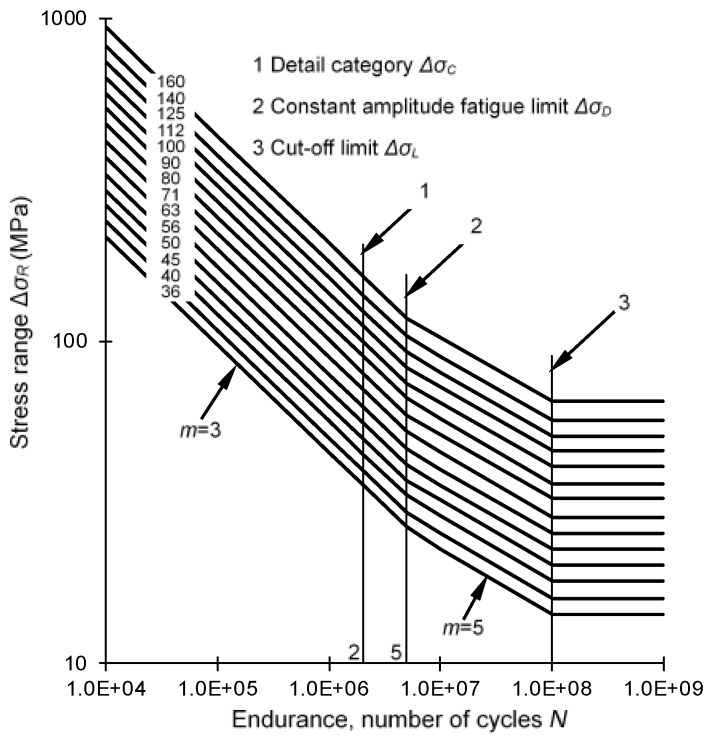
Fatigue strength curves in Eurocode 3.

Eurocode 3 provides the fatigue detail categories of welded orthotropic decks with either closed ribs or open ribs [7]. The S–N curves in Eurocode 3 adopt double slopes to consider the low-level stress cycles. As shown in Figure 1, when stress range is above the constant amplitude fatigue limit (CAFL) Δ*σ_D_*, the slope of the curves is −1/3; when stress range is below the CAFL, the slope of the curves is −1/5; and when stress range is below the variable amplitude fatigue limit (VAFL) Δ*σ_L_*, the curves become horizontal straight lines. Δ*σ_C_* is the detail category. Two types of weld details for OSDs, referring to rib-to-deck and the rib-to-rib details, respectively, will be investigated in this study. The S–N curve parameters for these welds are listed in Table 1.

For the nominal stress range spectrum, the fatigue strength curves are expressed as

(1a) $$\Delta \sigma_{R}^{3}N_{R}=\Delta \sigma_{C}^{3}\cdot 2\times 10^{6}=K_{C}\;\left(N\leq 5\times 10^{6}\right)$$

(1b) $$\Delta \sigma_{R}^{5}N_{R}=\Delta \sigma_{D}^{5}\cdot 5\times 10^{6}=K_{D}\left(5\times 10^{6}\leq N\leq 10^{8}\right)$$

where Δ*σ_R_* is the stress range; *N_R_* is the corresponding life in terms of number of cycles. In this study, the fatigue strength coefficient is defined as *K_C_* when Δ*σ_R_* is larger than Δ*σ_D_*, and *K_D_* when Δ*σ_R_* is smaller than Δ*σ_D_*. According to Equation (1a,b), the fatigue damage *D* induced by stress range *S* is

(2a) $$D=\frac{n}{N}=\frac{nS^{3}}{K_{C}}\left(\Delta \sigma_{D}\leq S\right)$$

(2a) $$D=\frac{n}{N}=\frac{nS^{5}}{K_{D}}\left(\Delta \sigma_{L}<S\leq \Delta \sigma_{D}\right)$$

where *n* is the number of *S* in the stress range spectrum, *N* is the fatigue life corresponding to stress range *S*. According to the Palmgren-Miner rule [15], the fatigue damage under variable amplitude loading can be calculated as

(3) $$D=\underset{\Delta \sigma_{D}\leq S_{i}}{\sum }\frac{n_{i}S_{i}^{3}}{K_{C}}+\underset{\Delta \sigma_{L}<S_{j}\leq \Delta \sigma_{D}}{\sum }\frac{n_{j}S_{j}^{5}}{K_{D}}$$

where *n_i_* is the number of stress range *S_i_* (larger than Δ*σ_D_*) and *n_j_* is the number of stress range *S_j_* (between Δ*σ_D_* and Δ*σ_L_*). Based on the equivalence principle, the variable amplitude stress range can be equivalent to a constant amplitude stress range, namely, the equivalent stress range. The expression of equivalent stress range *S_eq_* and corresponding number of cycles *N_c_* is [16]

(4) $$S_{eq}={\left(\frac{\sum_{\Delta \sigma_{D}\leq S_{i}}\frac{n_{i}S_{i}^{3}}{K_{C}}+\sum_{\Delta \sigma_{L}<S_{j}\leq \Delta \sigma_{D}}\frac{n_{j}S_{j}^{5}}{K_{D}}}{N_{c}/K_{D}}\right)}^{1/5}$$

(5) $$N_{c}=\underset{\Delta \sigma_{D}\leq S_{i}}{\sum }n_{i}+\underset{\Delta \sigma_{L}<S_{j}\leq \Delta \sigma_{D}}{\sum }n_{j}$$

Thus, the fatigue damage induced from *S_eq_* and *N_c_* can be written into

(6) $$D=\frac{N_{c}S_{eq}^{5}}{K_{D}}$$

For the fatigue reliability analysis based on monitoring data, the limit state function can be established as [13,16]

(7) $$G\left(X\right)=\Delta -e\cdot D=\Delta -e\cdot \frac{N_{c}S_{eq}^{5}}{K_{D}}=0$$

where Δ, the critical damage, represents the fatigue resistance, *e* is an error coefficient for field measurements [17,18]. The accumulated number of stress cycles *N_c_* is often treated as a deterministic variable and in the service years of *Y* it can be estimated by

(8) $$N_{c}\left(Y\right)=365\cdot Y\cdot ADSC$$

where *ADSC* is the average daily stress cycles, which can be determined using monitoring data. The parameters in Equation (7) can be treated as random variables to account for the uncertainties in both monitoring data and S–N curves. From existing literature [17,18,19,20], the lognormal distribution can be used to quantify the uncertainties in the error coefficient *e* [17,18], the critical damage Δ [19], and the fatigue strength coefficient *K_D_* [20]. The statistics of these variables are summarized in Table 2.

### 2.2. Probabilistic Model for the Equivalent Stress Range

Most studies indicated that the distribution of the *S_eq_* from monitoring data by SHMS are unimodal [21,22,23]. However, the authors’ most recent studies based on field measurements demonstrated that the *S_eq_* for certain OSD fatigue details is distributed multimodally. Hence, both the lognormal model and Gaussian mixture model (GMM) are employed to account for the actual distribution characteristics of the measured equivalent stress ranges.

GMM is one of the finite mixture distributions (FMD). The FMD has been used to quantify the uncertainty of the measured stress ranges in fatigue analysis of steel bridges [24,25]. For example, Ni et al. [24] fitted the FMDs to the measured stress range histograms of the Tsing Ma Bridge, in which several peaks are available which can be attributed to the highway traffic, the railway traffic, and the wind excitations [25]. In this study, the GMM is used to quantify the uncertainty of the measured daily equivalent stress ranges rather than the measured stress range histograms.

The probability density distribution of the GMM, which contains *M* Gaussian components, can be expressed as [26]

(9) $$f\left(x|\theta \right)=\overset{M}{\underset{i=1}{\sum }}w_{i}N\left(x|\mu_{i},\sigma_{i}^{2}\right)=\overset{M}{\underset{i=1}{\sum }}w_{i}\frac{1}{\sqrt{2\pi }\sigma_{i}}\cdot \exp\left[-\frac{{\left(x-\mu_{i}\right)}^{2}}{2\sigma_{i}^{2}}\right]$$

(10) $$\overset{M}{\underset{i=1}{\sum }}w_{i}=1$$

where $N\left(x|\mu_{i},\sigma_{i}^{2}\right)$ is the *i*th Gaussian component, of which the mean value and the variance are *μ_i_* and $\sigma_{i}^{2}$, respectively. *w_i_* denotes the weight of the *i*th component. The unknown parameter vector $\theta =\left(w_{1},\;w_{2},\;\ldots ,\;w_{M};\;\mu_{1},\;\mu_{1},\;\ldots ,\;\mu_{1};\;\sigma_{1}^{2},\;\sigma_{2}^{2},\;\ldots ,\;\sigma_{M}^{2}\right)$ is usually estimated by using the expectation maximization (EM) algorithm [27].

The Gaussian component number *M* should be priori information before estimating the unknown parameter vector ***θ*** with the EM algorithm. Although more Gaussian components can enable more accurate fitting results, subjective selections of the number of Gaussian component are inappropriate. Obviously, inadequate Gaussian components lead to inaccurate fitting results. On the other hand, excessive Gaussian components result in unnecessary complexity in the probability models. Hence, the Akaike information criterion (AIC) [28] and the Bayesian information criteria (BIC) [29] are employed to search the optimal Gaussian component number to achieve both accuracy and conciseness. The expressions of the AIC and BIC can be given as

(11) AIC=2m−lnL(x|M,θ)

(12) BIC=m×lnl−lnL(x|M,θ)

where *m* is the number of the unknown parameters; *l* is the length of the observed data sample; *L*(*x*|*M*, ***θ***) is the maximum value of the likelihood function of the fitted model. The Gaussian component number is the optimal number which produce lowest AIC or BIC.

### 2.3. Fatigue Reliability Estimation Methods

Based on the estimated probability distributions of *S_eq_* and the statistic information listed in Table 2, fatigue reliability can be obtained from either of the following two methods:

(1) **Method I**. An explicit formula of the fatigue reliability index *β* can be derived when the lognormal distribution is adopted for the daily *S_eq_*. For a variable *x* that follows a lognormal distribution, the probability density function is
  (13) $$f\left(x\right)=\{\begin{array}{c} \;\;\;\;\;\begin{array}{cc} 0 & \;\;\;\;\;\;\;\;x\leq 0 \end{array}\\ \begin{array}{cc} \frac{1}{\sqrt{2\pi }\sigma x}e^{-\frac{1}{2}{\left(\frac{\ln x-\mu }{\sigma }\right)}^{2}} & x>0 \end{array} \end{array}$$
  The mean value *μ*_ln*X*_ and the standard deviation *σ*_ln*X*_ of the variable ln*X* can be expressed as
  (14) $$\{\begin{array}{c} \mu_{\ln X}=ln\left(\frac{\mu_{X}}{\sqrt{1+\delta_{X}^{2}}}\right)\\ \sigma_{\ln X}=\sqrt{\ln\left(1+\delta_{X}^{2}\right)} \end{array}$$
  where *δ_X_* = *σ_X_*/*μ_X_*. Assuming that all the variables in Equation (7) are independent, the fatigue reliability index *β* can be defined as
  (15) $$\beta =\frac{\sum^{\text{​}}\mu_{\ln X_{i},R}-\sum^{\text{​}}\mu_{\ln X_{i},S}}{\sqrt{\sum^{\text{​}}\sigma_{\ln X_{i},R}^{2}+\sum^{\text{​}}\sigma_{\ln X_{i},S}^{2}}}=\frac{\mu_{\ln\Delta }+\mu_{\ln K_{D}}-\left(\mu_{\ln e}+\ln N_{c}+5\cdot \mu_{\ln S_{eq}}\right)}{\sqrt{\sigma_{\ln\Delta }^{2}+\sigma_{\ln e}^{2}+\sigma_{\ln K_{D}}^{2}+{\left(5\sigma_{\ln S_{eq}}\right)}^{2}}}$$
  where *μ*_ln*X*_ and *σ*_ln*X*_ denote the mean value and the standard deviation of ln*X* (herein, *X* = Δ, *e*, *K_D_* or *S_eq_*), respectively.
(2) **Method II**. The fatigue failure probability can be calculated by using the Monte Carlo method due to the difficulty in developing an explicit formula for the fatigue reliability index with the fitted GMM of *S_eq_*. Instead, the fatigue reliability index can be derived from the fatigue failure probability *p_f_*, which is simulated by using the Monte Carlo method. Therefore, the fatigue reliability index *β* is
  (16) $$\beta ={Ф}^{-1}\left(1-p_{f}\right)=-{Ф}^{-1}\left(p_{f}\right)$$
  where Φ^−1^(·) is the inverse CDF of standard normal distribution.

### 2.4. Proposed Outline for Fatigue Reliability Analysis

**Figure 2:**
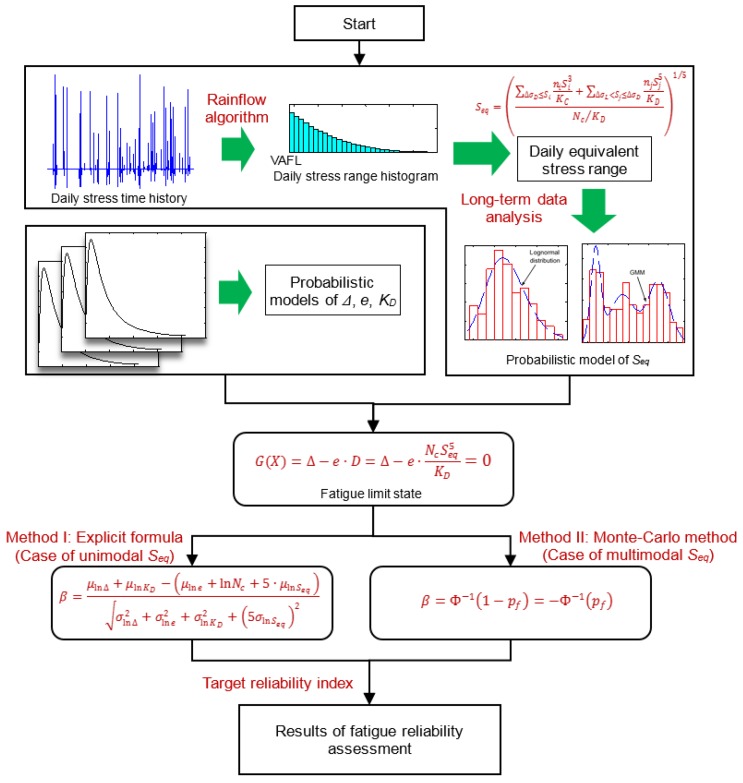
Outline of fatigue reliability assessment based on long-term strain monitoring.

Based on the above formulation, the proposed outline for fatigue reliability assessment based on long-term strain monitoring is summarized and presented in Figure 2.

## 3. Application on Runyang Suspension Bridge Based on Monitoring Data

### 3.1. Description of the Bridge and Strain Monitoring

**Figure 3:**
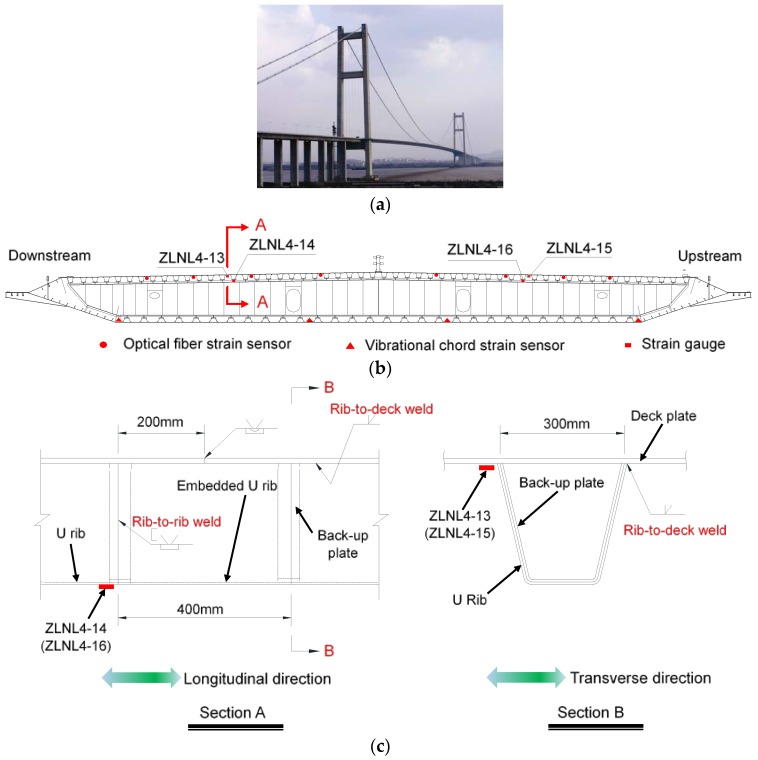
Layout of strain sensors at the mid-span of the RSB: (**a**) Overview of the RSB Prototype suspension bridge; (**b**) Mid-span cross section; (**c**) Orthotropic deck configuration.

Runyang Suspension Bridge (RSB), open to traffic in April 2005, is a single-span steel suspension bridge, as shown in Figure 3a. It has a main span length of 1490 m. The aerodynamically shaped closed box steel girder is 36.3 m wide and 3.0 m high. The material of the box girder is Q345 steel, with a yield strength of 345 MPa. The health monitoring system for the RSB was developed and installed for real-time monitoring of bridge responses under various environment actions and traffic loads [10]. Three types of strain sensors—i.e., optical fiber strain sensors, vibration chord strain sensors, and strain gauges—were installed on the mid-span section of the RSB’s steel box girder, as shown in Figure 3b.

The dynamic strain measurements collected by the strain gauges are processed and analyzed in this study. As shown in Figure 3c, gauges ZLNL4-13 and ZLNL4-15 are used for longitudinal strain measurements of the rib-to-deck weld details, while ZLNL4-14 and ZLNL4-16 are measuring transverse strains of the rib-to-rib weld details. The thicknesses of the deck plate and the U-shape rib are 14 mm and 6 mm, respectively. The rib-to-deck fillet welds were shop welded to obtain 100% penetration. U ribs were field spliced by butt welding in conjunction with two back-up plates and an embedded U rib segment. The thicknesses of the back-up plates and the embedded U rib are both 6 mm.

**Figure 4:**
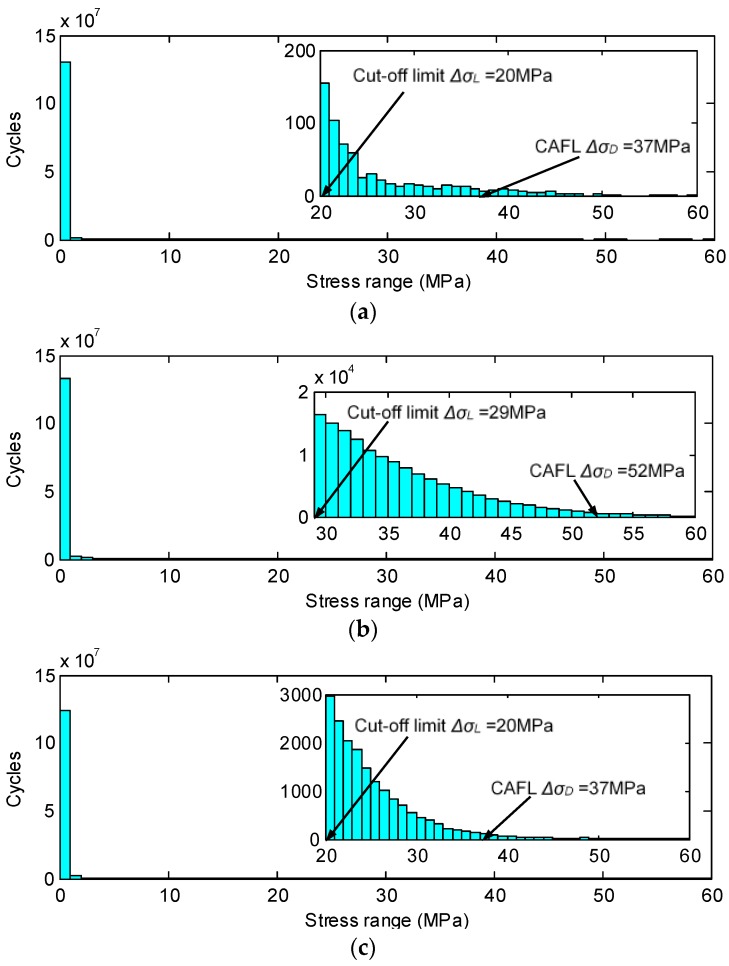

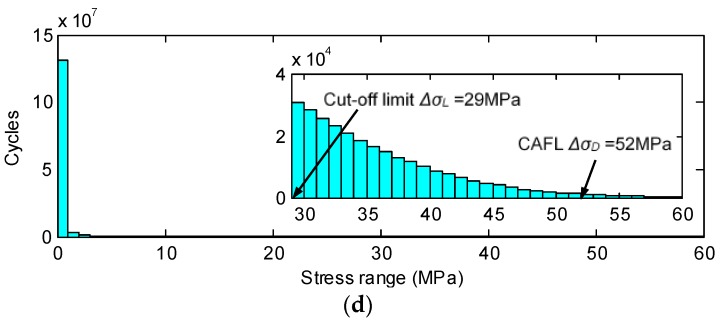
Stress range histograms: (**a**) ZLNL4-13; (**b**) ZLNL4-14; (**c**) ZLNL4-15; (**d**) ZLNL4-16.

To evaluate the long-term fatigue performance of the rib-to-deck and rib-to-rib weld details subject to operational traffic loadings, monitoring data collected during a total of 327 days in 2009 are used. The stress range histograms from the 327-day monitoring data are obtained by using the rain flow algorithm [30], as presented in Figure 4. It can be observed that small-amplitude stress ranges of less than 3 MPa dominate the histograms, which is a typical finding for fatigue details of highway steel bridges [3,19,24]. Table 3 lists the number of stress cycles in the histograms, which indicates that these details have a similar number of total cycles. The numbers of stress cycles, which are larger than the cut-off limit or the VAFL, only account for a small portion of the total cycles. Furthermore, the cycle number of the welded details in the downstream is less than that of the welded details in the upstream. This phenomenon may be attributed to the differences in the traffic volumes between upstream and downstream directions.

### 3.2. Probability Density Functions of S_eq_

**Figure 5:**
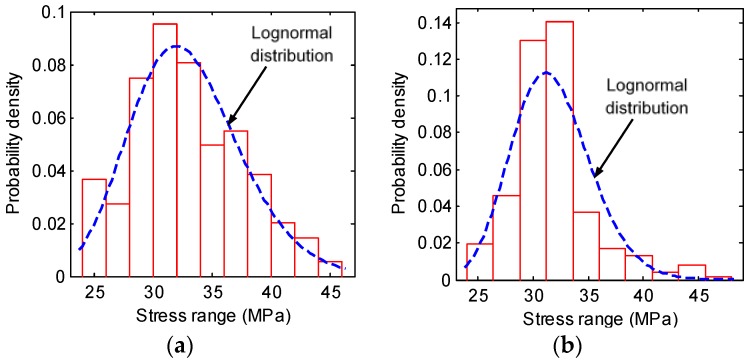
Probability densities of *S_eq_* of the rib-to-deck details: (**a**) ZLNL4-13; (**b**) ZLNL4-15.

**Figure 6:**
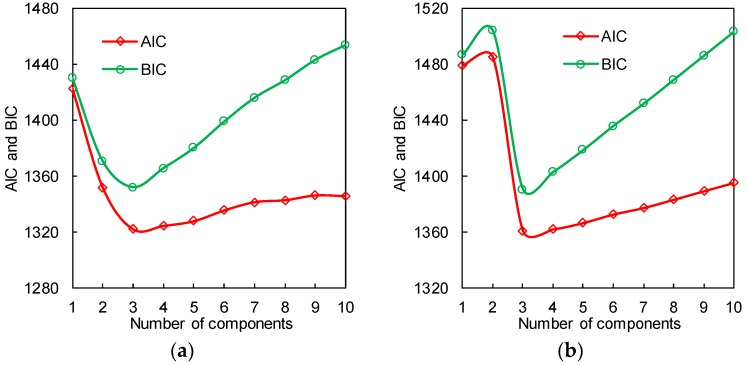
Criterions for GMM component determination: (**a**) ZLNL4-14; (**b**) ZLNL4-16.

As introduced in Section 2.2, the probability density function of *S_eq_* for the four selected weld details can be obtained using the lognormal distribution and the GMM. Firstly, daily stress range histograms can be derived from recorded stress time histories by the rain-flow counting. Then the *S_eq_* can be obtained using Equation (4). A total of 327 data points of *S_eq_* are obtained from 327-day measurements, which are further used to develop the probability density function of *S_eq_*. Figure 5 and Figure 6 plot the statistical histograms of *S_eq_* for the four details. As can be seen, for the rib-to-deck details, there is only one dominant peak. Therefore, the lognormal distribution can be fitted to these data points using the maximum likelihood method. The fitted probability density functions of *S_eq_* for the two rib-to-deck details are presented in Equations (17) and (18).

**Figure 7:**
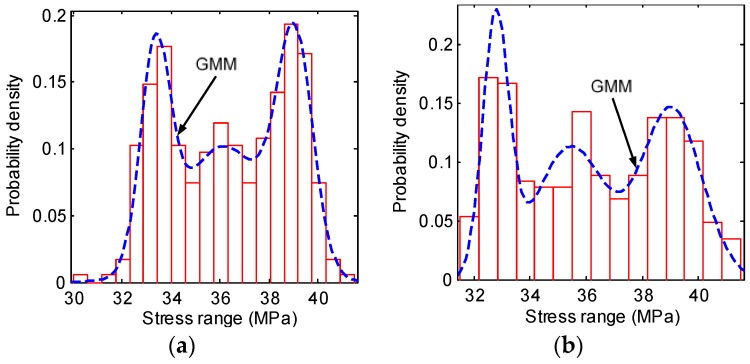
Probability densities of *S_eq_* of the rib-to-rib details: (**a**) ZLNL4-14; (**b**) ZLNL4-16.

For the rib-to-rib details, there are multiple peaks in the *S_eq_* histograms, and thus the GMM is used. The optimal number of Gaussian components is firstly determined by using the information criterions introduced in Section 2.2. Figure 7 shows the values of AIC and BIC with the number of Gaussian components varying from 1 to 10. It is observed that both the AIC and BIC are minimized when the number of Gaussian components is 3, meaning that the optimal number of Gaussian components for both ZLNL4-14 and ZLNL4-16 is 3. The estimated parameters are shown in Table 4.

(17) $$\mathrm{For}\;\mathrm{ZLNL}4-13,\;f\left(S_{eq}\right)=\begin{array}{cc} \frac{1}{\sqrt{2\pi }\times 0.142\times S_{eq}}e^{-\frac{1}{2}{\left(\frac{\ln S_{eq}-3.48}{0.142}\right)}^{2}} & S_{eq}>0 \end{array}$$

(18) $$\mathrm{For}\;\mathrm{ZLNL}4-15,\;f\left(S_{eq}\right)=\begin{array}{cc} \frac{1}{\sqrt{2\pi }\times 0.113\times S_{eq}}e^{-\frac{1}{2}{\left(\frac{\ln S_{eq}-3.45}{0.113}\right)}^{2}} & S_{eq}>0 \end{array}$$

### 3.3. Results of the Fatigue Reliability Assessment

With the statistic information listed in Table 2 and the probability density functions of *S_eq_* presented in Section 3.2, the fatigue reliabilities of the rib-to-deck details (with unimodal *S_eq_*) and the rib-to-rib details (with multimodal *S_eq_*) can be respectively calculated using Method I and Method II. It is noted that the times of the Monte Carlo simulation for rib-to-rib details is set to be 2 × 10^8^.

**Figure 8:**
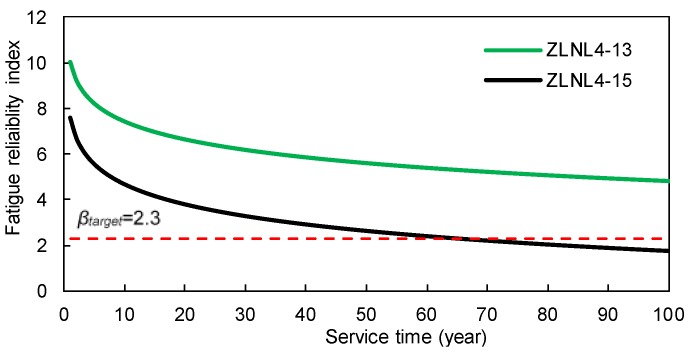
Time-dependent fatigue reliability indices of the rib-to-deck welds.

**Figure 9:**
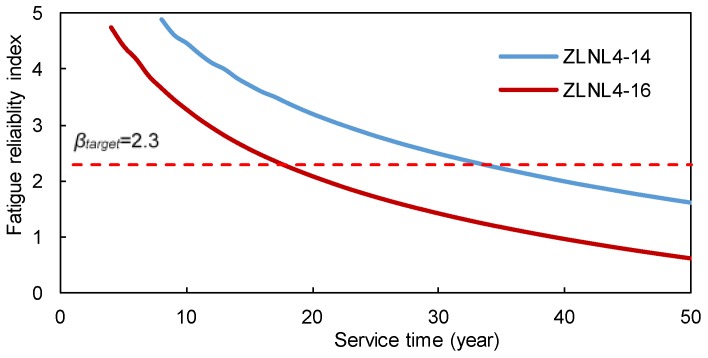
Time-dependent fatigue reliability indices of the rib-to-rib welds.

The time-dependent fatigue reliability indices are shown in Figure 8 and Figure 9. It is observed that the reliability indices are pretty high at the beginning of the service life of these welded details. However, as time goes on, the reliability indices decrease significantly. Figure 8 shows that during the first 10 years of the service life, the fatigue reliability indices of the rib-to-rib details cannot be obtained due to the extremely low failure possibility. According to the ISO 2394 [31], the target reliability index for a structural component with certain consequences of failure and moderate costs of safety measures is 2.3. As show in the figures, the service years for details of ZLNL4-14, ZLNL4-15 and ZLNL4-16 are 33, 65, and 18, respectively, when the fatigue reliability indices reach *β_target_*. Only the welded detail of ZLNL4-13 has fatigue reliability higher than *β_target_* during the entire 100-year service life. Results also indicate that for both the two types of weld details, the fatigue reliability index of the downstream detail is higher than that of the upstream detail. Besides, the reliability index of the rib-to-deck detail is higher than that of the rib-to-rib detail. The results suggest that during the service life the RSB, there are high levels of fatigue failure for welded details of the orthotropic deck. Hence, routine and in-depth field inspections are required to locate the potential fatigue cracks and make timely mitigation plans.

## 4. Conclusions

This study proposed a systematic time-dependent fatigue reliability assessment approach for welded details of OSDs using long-term strain monitoring data. Depending on whether the equivalent stress range is distributed unimodally or multimodally, the fatigue reliability can be calculated based on either an explicit formula or the Monte Carlo method. According to the proposed procedure, fatigue reliabilities of two types of welded details (i.e., rib-to-deck and rib-to-rib details) of an in-service long-span suspension bridge are investigated. The following conclusions can be made:

(1) Two probabilistic models, namely, the lognormal distribution and the GMM, are adopted to quantify uncertainties of the daily *S_eq_*. The lognormal distribution is more suitable for the unimodal *S_eq_* for rib-to-deck details. By contrast, the daily *S_eq_* for the rib-to-rib details can be represented by the GMM, which is composed of three Gaussian components.
(2) The results indicate that the reliability indices decrease significantly as the service life increases. During the 100-year service life, except for a rib-to-deck detail, other three welded details cannot meet the target fatigue reliability during the bridge’s 100-year service life.
(3) This study also reveals that the fatigue reliability indices of the downstream details are higher than those of the upstream details, which is probably due to the difference in the traffic volumes between upstream and downstream directions. Besides, the rib-to-deck details for the RSB have higher fatigue reliabilities than those of the rib-to-rib details.

The proposed approach can help bridge owners and practitioners make informed decisions regarding maintenance and repair of potential fatigue cracks.

## Figures and Tables

**Table 1:** S–N curves of Eurocode 3.

Description of Welds	Detail Category Δ*σ_C_* (MPa)	CAFL Δ*σ_D_* (MPa)	Cut-Off Limit Δ*σ_L_* (MPa)	Fatigue Strength Coefficient *K_D_*
Rib-to-deck	50	37	20	3.47 × 10^14^
Rib-to-rib	71	52	29	1.90 × 10^15^

**Table 2:** Statistic information of parameters.

Random Variable	Description	Distribution Type	Mean Value	COV	Source
*K_D_*	Rib-to-deck	Lognormal	3.47 × 10^14^	0.45	Zhao et al. [20], Eurocode 3 [7]
	Rib-to-rib	Lognormal	1.90 × 10^15^	0.45	
Δ	Critical damage	Lognormal	1.0	0.3	Wirsching [19]
*e*	Measurement error coefficient	Lognormal	1.0	0.03	Frangopol et al. [17,18]
*N_c_*	Accumulated number of stress cycles	Deterministic	-	-	SHM data

Δ and *e* are dimensionless. As defined in Equation (2), *K_D_* is related to the fatigue damage *D*, number of stress cycles *n*, and stress range *S* (unit: MPa). Therefore, the unit of *K_D_* is ${\mathrm{MPa}}^{5}\times \mathrm{cycle}$.

**Table 3:** Cycles of stress ranges.

Welded Details	Total Cycle Number	Cycle Number (Larger than Δ*σ_L_*)	Cycle Number (Larger than Δ*σ_D_*)
ZLNL4-13	1.319 × 10^8^	660.5	76.5
ZLNL4-14	1.384 × 10^8^	143,039	2537.5
ZLNL4-15	1.276 × 10^8^	17,620	826.5
ZLNL4-16	1.379 × 10^8^	270,087	5468

**Table 4:** GMM parameters for the equivalent stress range *S_eq_*.

Component *i*	ZLNL4-14			ZLNL4-16		
	*w_i_*	*μ_i_*	*σ_i_*^2^	*w_i_*	*μ_i_*	*σ_i_*^2^
1	0.306	39.0	0.54	0.262	32.8	0.23
2	0.236	33.3	0.37	0.346	35.4	1.48
3	0.458	36.1	3.23	0.392	39.0	1.15
